# Pasteurized waste milk *vs*. milk replacer at the same crude protein:metabolizable energy ratio with different energy sources (fat *vs*. lactose) to pre-weaning Holstein calves: Effects on growth performance, feeding behavior, and health

**DOI:** 10.1371/journal.pone.0317405

**Published:** 2025-01-16

**Authors:** Shahryar Kargar, Borhan Moradi, Meysam Kanani, Marzia Albenzio, Mariangela Caroprese, Mohammad Javad Zamiri, Ícaro Rainyer Rodrigues de Castro, Marcos Inácio Marcondes

**Affiliations:** 1 Department of Animal Science, School of Agriculture, Shiraz University, Shiraz, Iran; 2 Department of Animal Sciences, Washington State University, Pullman, Washington, United States of America; 3 Department of Agriculture, Food, Natural Resources and Engineering (DAFNE), University of Foggia, Via Napoli, Foggia, Italy; 4 Department of Animal Science, Universidade Federal de Viçosa (UFV), Viçosa, State of Minas Gerais, Brazil; University of Agriculture Faisalabad, PAKISTAN

## Abstract

The improved growth performance of calves at weaning results from an effective pre-weaning feeding strategy. The type and pasteurization process of liquid feed are among the most variable feeding practices affecting calves’ growth and health. In previous studies that compared waste milk (WM) *vs*. milk replacer (MR), little consideration has been given to the variations in chemical composition and feeding behavior between them, and there has been a lack of justification for the crude protein: metabolizable energy (CP:ME) ratio adopted. Hence, this study aimed to evaluate the effects of feeding pasteurized WM or MR differing in energy source (fat *vs*. lactose, respectively) with similar CP:ME ratio on intake, growth, feeding behavior, and health of newborn Holstein calves. Thirty-two male calves (4-d-old; 40.0 ± 0.58 kg BW) were assigned to the trial and randomly allocated to each liquid feed diet (WM or MR). Calves were housed in individual pens with free access to starter feed and fresh water. Calves were weaned on d 61 and assessed until d 101 as the postweaning period. WM-fed calves had greater total nutrient intake (DM, CP, EE, and ME), weight gain, final BW, skeletal growth parameters, and feed efficiency (d 30). Calves WM-fed sorted less against particles retained on the 2.36-mm sieve but more against particles retained on the sieve of 0.6 mm. In WM-fed calves, the sorting index decreased for feedstuff retaining on the bottom pan compared with MR-fed calves. Irrespective of the type of the liquid feed, all calves sorted for particles retaining on the sieve of 4.75 mm and the bottom pan, and against the particles that were retained on the sieves of 2.36- (MR-fed calves only), 1.18- and 0.6-mm. Starter feed nutrient intake and particle size intake from the sieves of 4.75-, 2.36-, and 1.18-mm increased in WM- *vs*. MR-fed calves. Eating rate and meal size but not meal frequency and length were greater in WM-fed calves, leading to higher pre- and post-weaning starter feed intake. Calves WM-fed spent less time eating and standing but more time ruminating and lying than MR-fed calves. Calves WM-fed had a lower likelihood of having elevated general appearance (score ≥2; hazard ratio = 2.79), diarrhea (score ≥3; hazard ratio = 1.35), and pneumonia (hazard ratio = 4.77). Calves WM-fed experienced shorter days with elevated general appearance, diarrhea, and pneumonia. Overall, feeding WM led to increased starter feed intake by boosting the eating rate and meal size, promoting greater growth than MR. Additionally, compared with MR, WM feeding increased time spent ruminating and lying and reduced susceptibility to diarrhea and pneumonia.

## Introduction

The effectiveness of any pre-weaning strategy is typically assessed by its impact on calf performance. Among the most variable management practices influencing calf health and growth are the type of liquid feed, waste milk (WM) or milk replacer (MR), and the pasteurization process [1–4]. Urie et al. [5] highlighted that morbidity and mortality rates in pre-weaned calves were much higher than the optimal morbidity (<25%) and mortality (<5%) rates [6]; therefore, there is a need for further improvement in the health and performance of dairy calves in the pre-weaning phase of life.

Waste milk is a common choice for feeding calves due to its availability and lower cost [2,4,7]. Furthermore, WM, as nonsaleable milk yielded in dairy farms, offers an economic advantage over commercial MR used in feeding young calves. Results of the study by Godden et al. [8] indicated that the economic benefit of feeding WM was $34/calf during the preweaning phase, compared with feeding MR. It differs significantly from most commercial MR in chemical composition, especially in energy, with higher fat content (>24% *vs*. 20% on a DM basis) and lower lactose (~35% *vs*. >42% on a DM basis) [2,3,9]. These differences in energy sources (fat *vs*. lactose) can affect milk osmolality (lower osmolality by fat *vs*. higher osmolality by lactose) [10–12], delay abomasal emptying [13], compromise gastrointestinal function [10], and impact calf health [9,11]. Urie et al. [5] found that calves fed ≤150 g fat/day had 3.1 times greater mortality odds before weaning than those fed ≥220 g fat/day, particularly if they experienced disease during this period. This emphasizes the need for a balanced approach when selecting milk type for calf feeding, linking nutrition directly to calf health outcomes. Waste milk also contains bioactive components like enzymes, hormones, and growth factors [2,14] but may include antibiotic residues [15–17]. However, studies suggest these residues do not affect starter feed intake or growth [17]. Instead, the compositional differences between WM and MR may influence health and growth, potentially leading to long-term benefits such as higher first-lactation milk yield, regardless of the milk type used [7,8,18].

Calf growth is primarily limited by protein and energy intake [19]. An optimal crude protein to metabolizable energy (CP: ME) ratio (~54 g CP/Mcal ME) is crucial to maximize weight gain and skeletal growth while avoiding over-conditioning [20]. Prior studies comparing WM and MR have often neglected the importance of this ratio [7,8]. This study standardizes the feeding level and CP: ME ratio, focusing on the predominant variance in energy sources: fat in WM and lactose in MR.

Feeding behavior is regulated by energy homeostasis and health status, influenced by the type of energy provided (fat *vs*. lactose) [21]. In young calves, glucose and fatty acids (FA) are primary energy sources, with metabolic signals from glucose utilization (glucostatic theory) and FA oxidation (lipostatic theory) playing key roles in feed intake regulation [22]. For instance, calves fed high-lactose MR showed increased visits to automatic feeders during weaning, suggesting hunger-related behavior without affecting starter feed intake [12]. The mechanisms by which energy sources in liquid feed (WM *vs*. MR) affect starter feed intake remain unclear. Thus, examining the feeding behavior of calves fed WM *vs*. MR, differing in energy sources but with similar CP ratios, may clarify how liquid feed type influences feed intake. Thus, this study aimed to assess the effects of feeding WM or MR, differing in energy sources but with similar CP ratios, on the performance, feeding behavior, and disease susceptibility of pre-weaning Holstein calves. We hypothesized that feeding WM would enhance growth performance, potentially through increased nutrient intake or improved health status.

## Materials and methods

### Animals, treatments, and management

This trial was conducted from October 2, 2019 until January 15, 2020, at the Foudeh-Sepahan Agriculture and Animal Husbandry, Isfahan, Iran. All animal procedures were executed according to the Iranian Council of Animal Care [23] recommendations (IACUC #9731916). Calves were borrowed from a commercial dairy farm and no calf was euthanized in this study by the authors. Several criteria were used to select the calves for the experiment. The herd veterinarian used the vigor scoring system to check each calf for health status at birth [24], which accesses visual appearance, initiation of movement, general responsiveness, oxygenation and rates of heart beat and respiration. Calves with diarrhea, fever, physical impairments, failure of suckle, and other health-related problems were excluded from the trial. Holstein male calves weighing between 35 and 45 kg at birth, with 24-h total blood protein level >5.5 g/dL, were randomly distributed into two treatments. Thus, 32 calves (n = 16 per treatment; 4 d of age; BW = 40.0 ± 0.58 kg; blood total protein = 6.0 ± 0.16; dam parity = 2.7 ± 0.20; mean ± SE) were placed in a naturally ventilated barn with individual pens (2.9 m × 1.1 m × 1.8 m; length × width × height). Wheat straw bedding was changed every other day, and daily manure removal was done to maintain the pens properly clean and dry.

The calves were fed 5 kg pasteurized (at 60°C for 90 min; IG-PLUS; Shirmack Pasteurizer, Shirmack Livestock Engineering Group, Isfahan, Iran) colostrum (Brix value ≥ 22.0%) within the first 2-h after birth (3.0 kg) using a nipple bottle and another meal at 6-h following the first feeding (2.0 kg). On d 2 and 3 of life, all calves consumed 5 kg of pasteurized transition milk (at 60°C for 90 min; 4 kg) in steel buckets in 2 equal meals (at 09:00 and 17:00).

Starting from d 4, half of the calves (16 in total, randomly selected) received a diet of pasteurized WM (non-saleable milk; heated at 67.5°C for 30 minutes) with a set temperature of 40 ± 1.0°C. This whole milk contained a specific percentage of CP (23.4 ± 0.13%) and fat (24.8 ± 0.30%; mean ± SE), had antibiotic residues (milk from mastitis cows undergoing treatment), and was standardized for 11% DM. The feeding schedule for these calves was as follows: 6.0 kg/d from d 1 (the 4th d of their life) until d 53, 5.0 kg on d 54, 4.0 kg/d from d 55 to d 56, 3.0 kg/d from d 57 to d 58, 2.0 kg on d 59, and 0.5 kg for the morning feeding on d 60 of the trial. The other 16 calves were given a diet of MR (Imperial; Novin Roshd Shahran Foudeh, Isfahan, Iran) with 22% CP and 17% fat. Both WM and MR were provided in steel buckets and administered in two equal meals, at 09:00 and 17:00.

Each day, WM was collected, placed in a separate refrigerator, and then pasteurized with a blender dedicated solely to this purpose. This milk was fed to the calves, and a record of any milk they refused was kept. Additionally, calves had free access to mashed starter feed and fresh water, both provided in steel buckets (Table 2). On d 61, calves were weaned and continued in the trial until d 101, during which they were individually housed in pens. The procedures remained like the preweaning period, except that milk was no longer part of their diet. It is essential to state that the calf keepers and trial staff were aware of the treatment distribution at different stages throughout the experiment.

### Sampling and analyses

Blood samples from the jugular vein were taken 24-h following the first colostrum feeding in collection tubes (BD Vacutainer, Franklin Lakes, NJ, USA) containing spray-coated silica to quantify the serum total protein with a commercially handheld clinical refractometer (ATA-2771; Atago Co. Ltd., Tokyo, Japan). The average serum total protein (± SE) was 6.07 ± 0.17 and 5.93 ± 0.14 g/dL in WM or MR treatment groups, respectively (*P* = 0.396).

Pooled pasteurized WM and MR were sampled daily (two samples/d; one sample at each feeding time), stored at 4°C, and transferred to the Central Milk Testing Laboratory of the farm for determination of DM, protein, fat, and lactose concentrations using an infra-red analyzer (MilkoScan 134 BN; Foss Electric, Hillerød, Denmark) as well as pH measurement using a portable pH-meter (model AZ8685; AZ Instrument Corp., Taichung, Taiwan; Table 1). The values on nutrient composition were used to calculate the daily nutrient intake.

**Table 1:** Daily nutrient composition of pasteurized waste milk or milk replacer^1^ fed to newborn Holstein calves.

Chemical composition	Treatments	
	WM	MR
Dry matter (DM)	10.23 ± 0.90	10.86 ± 0.33
CP, % of DM	23.37 ± 1.20	21.09 ± 0.10
Fat, % of DM	24.78 ± 2.78	16.72 ± 0.97
Lactose, % of DM	34.32 ± 3.46	43.21 ± 0.35
ME, Mcal/kg of DM	5.04 ± 0.16	4.61 ± 0.04
CP:ME ratio, g/Mcal	46.37 ± 0.26	45.75 ± 0.10
pH	6.76 ± 0.12	6.50 ± 0.09

WM = waste milk; MR = milk replacer; CP = crude protein; ME = metabolizable energy; pH = potential of hydrogen.

^1^Milk replacer (Imperial; Novin Roshd Shahran Foudeh, Isfahan, Iran) contained skim milk, whey powder, whey protein concentrate, vegetable fat, vitamins (contained per kg of product: 25000 IU of vitamin A, 5000 IU of vitamin D_3_, 150 mg vitamin E, 8 mg vitamin B_1_, 10 mg vitamin B_2_, 6 mg vitamin B_6_, 300 mg vitamin C, 2 mg vitamin K, 300 mg choline chloride, and 200 mg biotin), minerals (contained per kg of product: 80 mg of Zn, 70 mg of Mn, 100 mg of Fe, 1 mg of I, 0.4 mg Se, and 0.6 mg Co), and saccharomyces cerevisiae (10 mg per kg of product). Milk replacer contained (on DM basis) 22% CP, 17% fat, 0.4% crude fiber, 8% ash, 0.9% calcium, and 0.6% phosphorous.

Samples of starter feed (n = 11; pooled within trial period) and calf refusals (n = 10/calf; pooled by calf within treatment) were collected every 10 days over the trial period to measure the DM and nutrient composition. Specifically, refusal samples for each calf within their respective treatment were taken immediately before the morning feeding. Dry matter content was evaluated using a forced-air oven by drying at 100°C for 24 h (method 925.40) [25]. The samples were ground to pass a 1-mm screen in a Wiley mill (Ogawa Seiki Co., Ltd., Tokyo, Japan) and analyzed in duplicate for CP (Kjeltec 1030 Auto Analyzer, Tecator, Höganäs, Sweden; method 955.04) [25], ether-extract (EE; method 920.39) [25], crude ash (method 942.05) [25], and neutral detergent fiber (NDF) using a heat-stable α-amylase (100 μL/0.5 g of sample) and sodium sulfite [26] (Table 2).

**Table 2:** Ingredients, chemical composition (% of DM unless otherwise noted), and particle size distribution of the basal starter feed.

Ingredient composition	Value
Alfalfa hay	10.00
Wheat bran	7.10
Corn grain, ground	47.00
Barley grain, ground	14.10
Soybean meal	18.80
Calcium carbonate	1.20
Vitamin and mineral mixture^1^	0.57
Salt	0.47
Magnesium oxide	0.38
Bentonite	0.38
Chemical composition	
Dry matter (DM)	91.92
Crude protein (CP)	19.10
Neutral detergent fiber (NDF)	19.21
Ether extract (EE)	3.24
Ash	5.73
Calcium	0.74
Phosphorous	0.43
Metabolizable energy (ME),^2^ Mcal/kg of DM	3.01
CP:ME ratio, g/Mcal	63.46
Particle size distribution (% of DM retained on sieves)	
4.75 mm	19.65
2.36 mm	15.35
1.18 mm	35.30
0.6 mm	20.01
Pan	9.69
pef_>2.36_^3^	0.35
pef_>1.18_^3^	0.70
pef_>0.6_^3^	0.90
peNDF_>2.36_^3^	6.71
peNDF_>1.18_^3^	13.50
peNDF_>0.6_^3^	17.32
Xgm,^4^ mm	1.80
SDgm,^5^ mm	1.54

^1^Contained per kilogram of the supplement: 975,000 IU of vitamin A, 750,000 IU of vitamin D, 1,800 IU of vitamin E, 143 g of Zn, 76 g of Mn, 48.6 g of Cu, 19.5 g of Se, 18.4 g of Fe, 8 g of Ca, and 1.3 g of Co.

^2^ME = TDN × 0.04409 × 0.82; calculated according to NRC [27].

^3^pef_>2.36, 1.18, and 0.6_ = physical effectiveness factor determined as the proportion of particles retained on 2 (4.75 and 2.36 mm), 3 (4.75, 2.36, and 1.18 mm), and 4 (4.75, 2.36, 1.18, and 0.6 mm) sieves; peNDF_>2.36, 1.18, and 0.6_ = physically effective neutral detergent fiber (NDF) determined as NDF content of starter feed diets multiplied by pef_>2.36, 1.18, and 0.6_, respectively.

^4^Geometric mean particle size (Xgm), calculated according to ASAE [30] method S424.1.

^5^Geometric standard deviation of particle size (SDgm), calculated according to ASAE [30] method S424.1.

To determine particle size distribution, extra samples of the basal starter feed (n = 11; one sample every 10-d during the trial period) and individual refusals (n = 5; pooled by calf every 10 d over the trial period from d 51 through d 101 of the trial period) were taken and screened with a 4-screen (4.75, 2.36, 1.18, and 0.6 mm) particle separator (Model 120; Automatic Sieve Shaker, Techno Khak, Khavaran, Tehran, Iran) into five fractions [28,29]. To measure the particle size, 200 g of each sample (basal starter or orts) was placed on the top screen and the stack of sieves was shaken for about 10 min so that the distribution of feed materials remained without any changes [28,29]. After sifting, the DM content of each separated fraction was determined by drying at 100°C for 24 h using a forced-air oven (method 925.40) [25]. The physical effectiveness factor (pef) was calculated as the DM proportion of particles retained on sieves two (pef _> 2.36_), three (pef _> 1.18_), and four (pef _> 0.6_). The physically effective NDF on sieves two (peNDF _> 2.36_), three (peNDF _> 1.18_), and four (peNDF _> 0.6_) was calculated by multiplying the NDF concentration of the feed by the fraction of pef_>2.36_, pef_>1.18_, and pef_>0.6_, respectively. The geometric mean particle size of the starter feed diet (Table 2) was calculated according to ASAE (method S424.1) [30].

### Nutrient intake and growth indicators

To determine calf individual feed intake, starter feed offered and refusals (taken daily at 10:00 h before delivery of fresh starter feed) were weighted and recorded daily using a calibrated electronic scale (SF-400; Etminan Co., Tehran, Iran). Starter feed was offered at a rate that allowed at least 10% refusals; therefore, daily starter feed intake was adjusted as the calf grew. The intake of each nutrient originating from liquid feeds (WM and MR) and starter feed diet was used to compute total nutrient intake.

Body weight was evaluated with an electronic scale (WLC; Etemad Co., Tehran, Iran) at birth, before the morning feeding at the beginning (d 1) of the trial, and every 10-d thereafter, and the average daily gain (ADG; g of BW/d) was calculated as the difference between BW taken at 10-d intervals divided by 10. Feed efficiency was calculated as a gram of weight gain divided by total DM intake (milk DM + starter feed DM). Skeletal measurements, including the heart girth, withers height, body length, hip height, and hip width (were measured at the beginning (d 1) and end of the trial (d 101), and the skeletal gain (d 1 to 101) was calculated accordingly.

### Feed sorting and chewing activity

To evaluate if calves were performing sorting in the starter feed diet, 1 sorting value was created per calf per 10-d starting on d 51 through d 101 of the trial for each particle size fraction. Sorting activity was estimated as the ratio of real intake to the expected intake for particles retained on each sieve [31]. The predicted intake of an individual fraction was calculated as the total diet DM intake multiplied by the DM percentage of that fraction in the fed starter feed, with values of 100%, <100%, and >100% indicating no sorting, sorting against, and sorting for each particle size, respectively.

Calves were visually monitored (every 5 min) by two trained observers to capture the eating, ruminating, resting, drinking, non-nutritive oral behaviors (NNOB; when the calf licked any surface, tongue rolling, etc.), standing, and lying for a period of 8 h (from 10:00 to 18:00), once per three succeeding days before weaning (d 57–59 of the trial) in addition to, once per 3 succeeding days after weaning (d 87–89 of the trial) [32]. One observation (at least) of eating activity occurring after at least 5 min without eating was considered a period of eating. Meal frequency was defined as the number of bouts for 8 h. The meal length (min/meal) was computed as the time from the beginning of the first feeding event until an interval between events and averaged for each calf. Inter-meal intervals (min) were calculated from the end of one feeding event to the beginning of the next one and averaged for each calf. The speed of eating (g starter feed DM/min) was computed as the total amount of starter feed DM consumed during a period of 8 h divided by the time dedicated to eating and averaged for each calf. The meal size (g starter feed DM/meal) was the total amount of starter feed DM intake consumed during each meal. The rumination pattern was calculated using the same procedure.

### Health

During the milk feeding period (d 1 to 61), the calves were daily evaluated for health status based on their appetite and desire to consume the liquid (WM or MR) and starter feeds together with their general appearance by a veterinarian, blinded to the treatments, according to a referenced standard [33]. The fecal score was recorded daily (before the morning feeding) for physical shape and consistency (1 = normal; 2 = soft to loosen; 3 = loose to watery; 4 = watery, mucous, slightly bloody; and 5 = watery, mucous, and bloody) while calves were in individual pens. General appearance scores were assigned on a 1-to-5 scale: 1 = normal and alert; 2 = ears drooped; 3 = head and ears drooped, dull eyes, slightly lethargic; 4 = head and ears drooped, dull eyes, lethargic; and 5 = severely lethargic. The fecal score was categorized as the number of days with a fecal score ≥3, and general appearance was categorized as the number of days with a general appearance score ≥2. These classifications were denoted as days with abnormal fecal scores and general appearance, respectively [32,34]. Calves with fever (≥39.4°C as fever threshold) and abnormal general appearance, fecal score, or cough were examined by the veterinarian, unaware of the treatments, for confirmation of diarrhea or pneumonia.

Calves with diarrhea or pneumonia were treated following the standard practices at the Foudeh-Sepahan Agriculture and Animal Husbandry (Isfahan, Iran). Calves with diarrhea received a water-based oral rehydration salt solution [ORS; containing 500 mg dextrose, 250 mg sodium chloride, and 250 mg sodium bicarbonate per g; 4 L/d (10 g ORS/L) per calf in 2 meals of equal volume (at 12:00 and 20:00 h)] for five consecutive days; Rooyan-e-Isfahan Co., Isfahan, Iran) and neomycin (500 mg neomycin sulfate per bolus; 2 boluses/d per calf before milk feedings for 5 consecutive days; Iran Pharmaceutical Products Co., Semnan, Iran). Non-responding individuals received an intravenous liquid therapy using sodium bicarbonate solution (1.3%; 1.5 L/calf; Iran Pharmaceutical Products Co.), sugar and salt solution (dextrose 3.33% + sodium chloride 0.30%; 1 L/calf; Shahid Ghazi Pharmaceutical Co., Tabriz, Iran) with a single dose vitamin AD_3_E (containing 50000 IU vitamin A, 10000 IU vitamin D_3_, and 20 mg vitamin E per mL; 4 mL/calf; Rooyan Darou Co., Semnan, Iran) + B_12_-P complex (containing 0.05 mg cyanocobalamin and 125 mg sodium-α-oxybenzylphosphinicom per mL; 4 mL/calf; Razak Laboratories Co., Karaj, Iran) injection. Whenever blood was seen in diarrhea, calves were treated with enrofloxacin (5%; 4 mL/calf for five consecutive days; Rooyan Darou Co.) and flunixin (5%; 4 mL per calf on the first day of treatment protocol; Razak Laboratories Co.) with a single dose vitamin B_1_ (containing 200 mg thiamine hydrochloride per mL; 4 mL per calf on the first day of treatment protocol; Rooyan Darou Co.).

To treat pneumonia, calves were medicated with enrofloxacin (5%; 5 mL/calf for five consecutive days; Rooyan Darou Co.) and flunixin (5%; 4 mL/calf on the first day of treatment protocol; Razak Laboratories Co.) with a single dose multi-vitamin (containing 30 MIU vitamin A, 8 MIU vitamin D_3_, 16 KIU vitamin E, 2 g vitamin B_1_, 2 g vitamin B_2_, 20 g vitamin B_3_, 5 g vitamin B_5_, 2 g vitamin B_6_, 10 mg vitamin B_12_, 10 g vitamin C, 20 mg biotin, 30 g methionine, and 20 g lysine per L; 4 mL/d/calf on the first day of treatment protocol; Rooyan Darou Co.). Non-responding calves received the above-mentioned treatment plus pantries (containing 200 mg trimethoprim and 200 mg sulfamethoxazole per mL; 5 mL/calf for five consecutive days; Makian Daru Co., Tehran, Iran).

### Statistical analyses

Pre-trial power analysis for sample size calculation was performed using ADG data according to recently published literature [1,35]. From the power test analysis, using α = 0.05 and power = 0.80, the projected sample size was 12 calves per treatment group. Therefore, a total of 32 calves was found sufficient to get a significant result with adequate power (POWER PROC; SAS, version 9.4; SAS Institute Inc., Cary, NC, USA).

Data on nutrient intake (d 1 to d 61 and d 1 to d 101), BW (d 1 to d 101), ADG (d 1 to d 101), feed efficiency (d 1 to d 101), skeletal measurements (d 1 to d 101), feeding and chewing behavior, sorting activity and actual particle size fraction intake of DM (d 51 to d 101), were subjected to ANOVA using the MIXED procedure with times (1- or 10-d period) as repeated measures. The calf was considered as a random effect, and treatment (T; the effect of feeding WM *vs*. MR), period (P; 1- or 10-d period), and T × P as fixed effects. Initial, weaning, and final BW data were analyzed using the same model without the P effect. Each fraction was tested for sorting activity considering a difference from 100% using the t-test procedure to test whether sorting happened. Several variance-covariance structures were tested, and the auto-regression structure (type 1) with minimized Bayesian information criterion was accordingly modeled. The SLICE statement of the MIXED procedure (PROC MIXED) of SAS was used to perform partitioned analyses of the least squares means for interaction between T and P when required. Data were reported as the least squares mean and considered significant if *P* ≤ 0.05; a tendency was reported if 0.05 < *P* ≤ 0.10.

Models for the occurrence of general appearance (≥2), diarrhea (≥3), and pneumonia were evaluated during the pre-weaning period by logistic regression using a binomial distribution in the GLIMMIX procedure. The odds ratio was used to compare the likelihood for calves in each treatment group to experience any event. The number of days with general appearance (≥2), frequency and duration of diarrhea (≥3) or pneumonia, and administration of medication were tested (Poisson distribution) using the GENMOD procedure. Based on that, a survival analysis model was performed using the Cox Proportional Hazards Regression Analysis, describing the probability (with a 95% confidence interval) of survival for the calves during the days of the experiment receiving the different milk supplements evaluated (hazard ratios). The survival was modeled as a function of calf age, with d 1 as the date of the calf’s birth up to weaning (60 d of life) using a Kaplan-Meier survival curve.

## Results

### Intake and growth

**Fig 1:**
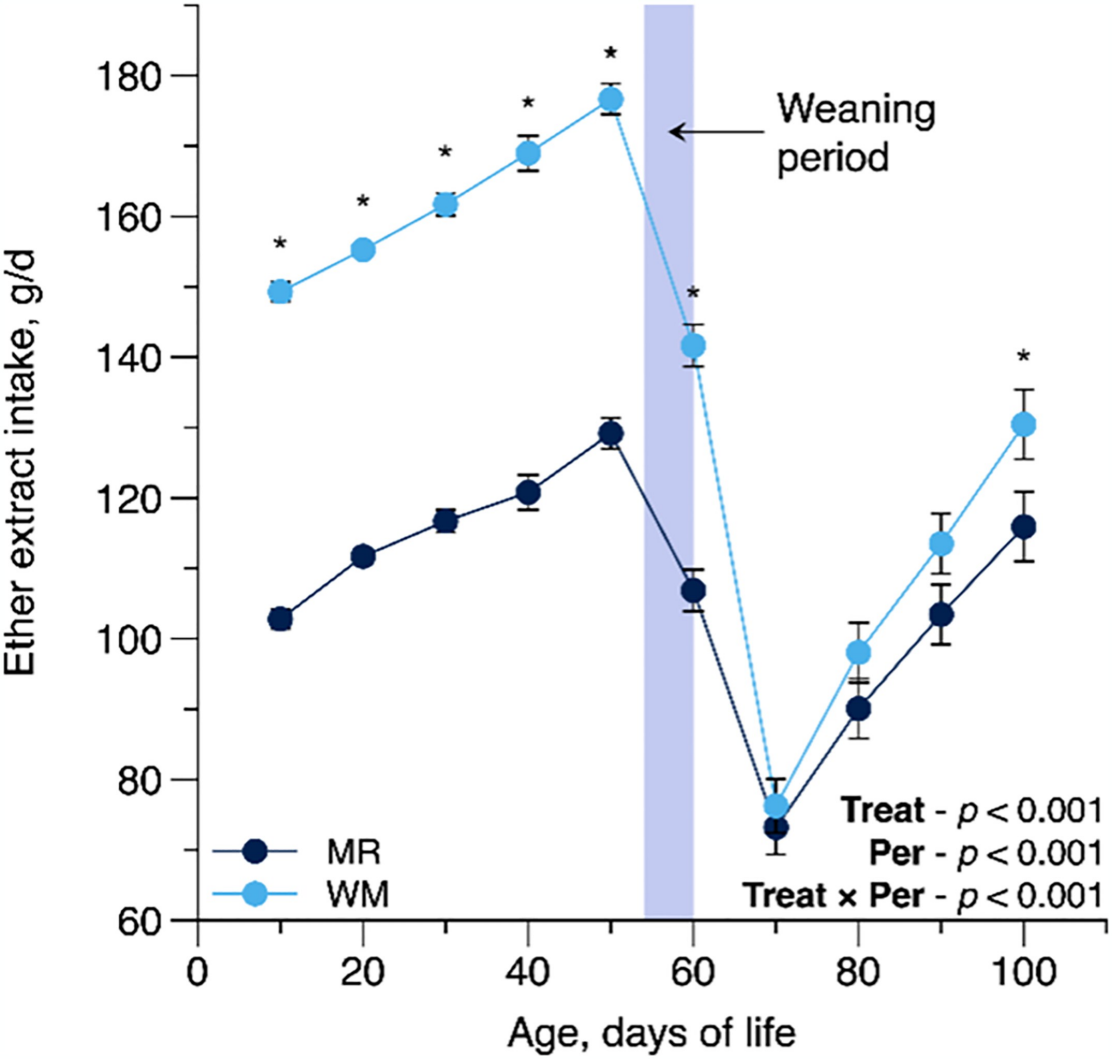
Interaction panel of Treat × Period effect (T × P) for ether extract intake (g/d) as influenced by feeding pasteurized waste milk (WM) or milk replacer (MR) to newborn Holstein calves (n = 16 per treatment). For each time point, * denotes a significant difference at *P* ≤ 0.05. Error bars represent the standard error of the mean.

A treatment-by-period interaction (T × P effect) influenced ether extract intake (*P* = 0.001) that was greater in WM-fed calves when compared to MR-fed calves, mostly during the pre-weaning period (Table 3 and Fig 1). In contrast, by design, lactose intake was consistently higher for MR-fed calves than WM-fed calves during the pre-weaning period since MR naturally has more lactose than raw milk. Similarly, a trend in the interaction between treatment and period was observed for SI (*P* = 0.052; Fig 3A), showing a pattern consistent with the ether extract intake. Further, WM-fed calves presented greater intakes for the starter feed as a function of BW, DM as a function of BW, CP, and ME when compared with MR-fed calves. The treatments did not affect total DMI between the two groups (Table 3).

**Table 3:** Nutrient intake and growth performance as influenced by feeding pasteurized waste milk or milk replacer to newborn Holstein calves (n = 16 per treatment).

Item	Treatments (T)		SEM	*P*-value		
	MR	WM		T	Period (P)	T × P
Initial BW, kg	40.09	39.91	0.589	—	—	—
SI, g/d	1459.34	1685.54	53.800	0.007	0.001	0.052
SI_BW, %	3.636	4.053	0.139	0.041	0.001	0.372
MI, g/d	601.85	573.68	2.088	—	—	—
Total DMI, g/d	1820.44	1965.80	64.826	0.113	0.001	0.449
DMI_BW, %	4.525	4.918	0.135	0.048	0.001	0.203
CPI, g/d	353.69	390.17	12.029	0.030	0.068	0.588
NDFI, g/d	280.20	323.63	10.329	0.007	0.001	0.052
EEI, g/d	107.08	137.19	2.057	0.001	0.001	0.001
MEI, Mcal/d	6.04	6.62	0.189	0.039	0.001	0.292
ADG, g/d	812.50	928.75	22.572	0.001	0.001	0.046
BW, kg	75.61	81.20	1.165	0.001	0.001	0.023
FE, g/g	0.495	0.545	0.009	0.001	0.001	0.001
Hip height, cm	90.04	91.89	0.289	0.001	0.001	0.001
Hip width, cm	17.54	18.25	0.095	0.001	0.001	0.001
Body length, cm	54.13	55.42	0.147	0.001	0.001	0.018
Body barrel, cm	113.10	115.58	0.772	0.030	0.001	0.001
Withers height, cm	87.98	90.16	0.301	0.001	0.001	0.001
Heart girth, cm	97.69	101.52	0.465	0.001	0.001	0.001

MR = milk replacer; WM = waste milk; SI = starter intake; SI_BW = starter intake % of initial body weight; MI = milk intake; DMI = dry matter intake; DMI_BW = dry matter intake % of initial body weight; CPI = crude protein intake; NDFI = neutral detergent fiber intake; EEI = ether extract intake; MEI = metabolizable energy intake; ADG = average daily gain; BW = body weight; FE = feed efficiency; SEM = standard error of the mean.

**Fig 2:**
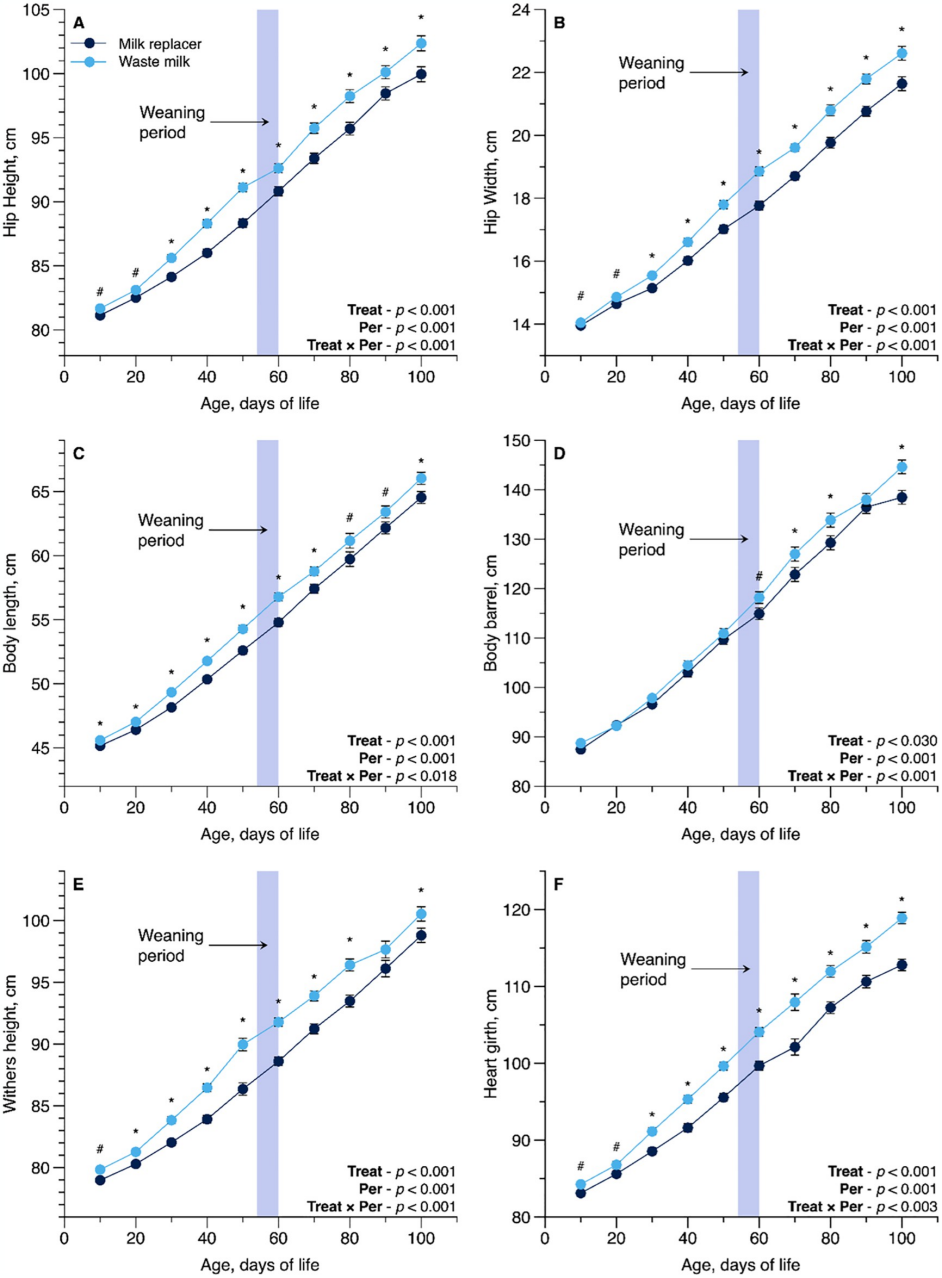
Interaction panel of Treat × Period effect (T × P) for body measurements as influenced by feeding pasteurized waste milk (WM) or milk replacer (MR) to newborn Holstein calves (n = 16 per treatment). For each time point, * denotes a significant difference at *P* ≤ 0.05, and ^#^ denotes a tendency at 0.05 < *P* ≤ 0.10. Error bars represent the standard error of the mean.

**Fig 3:**
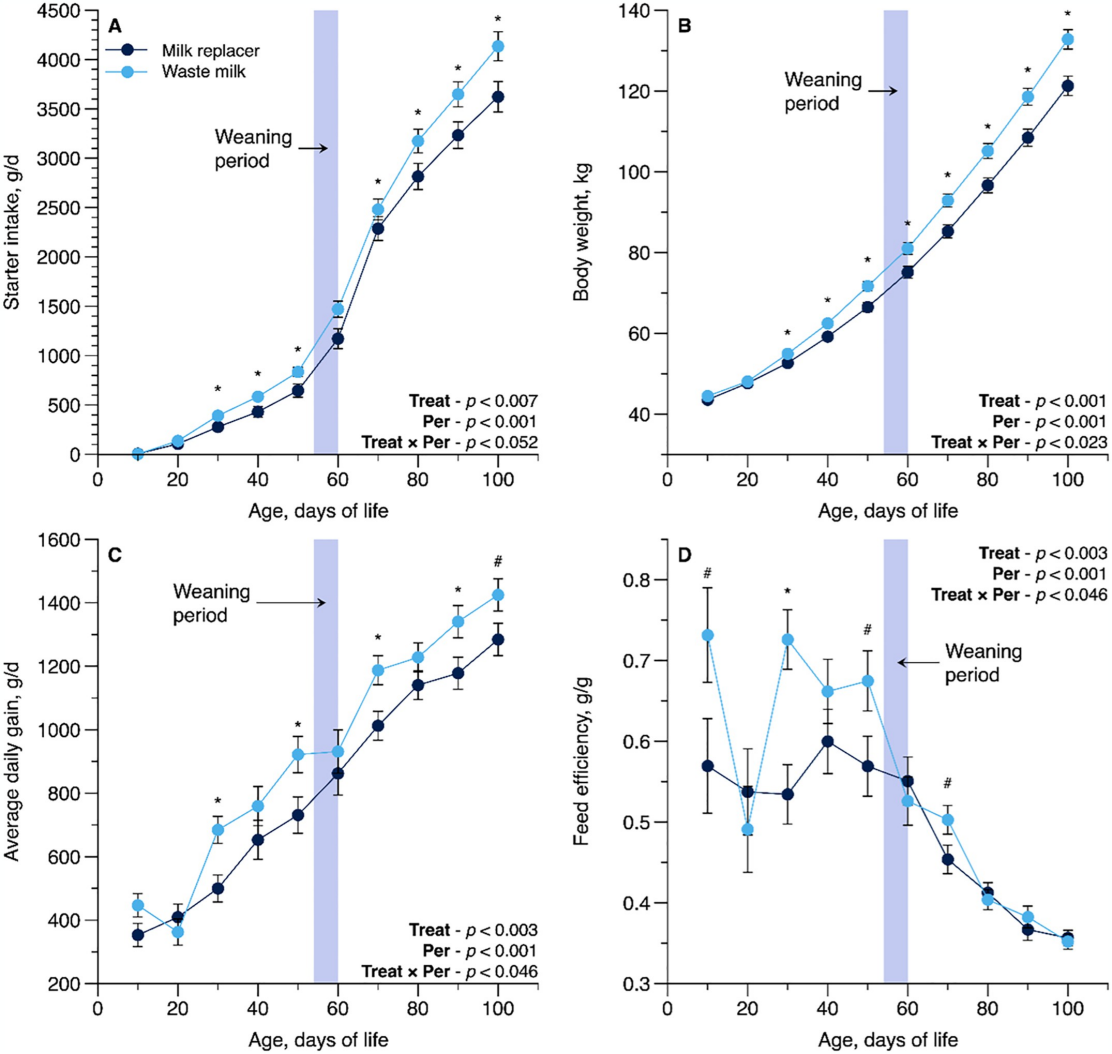
Interaction panel of Treat × Period effect (T × P) for starter intake (g/d), body weight (kg), average daily gain (g/d), and feed efficiency (g/g) as influenced by feeding pasteurized waste milk (WM) or milk replacer (MR) to newborn Holstein calves (n = 16 per treatment). For each time point, * denotes a significant difference at *P* ≤ 0.05, and ^#^ denotes a tendency at 0.05 < *P* ≤ 0.10. Error bars represent the standard error of the mean.

All skeletal growth measurements showed a T × P interaction effect including hip height (*P* = 0.001; Fig 2A), hip width (*P* = 0.001; Fig 2B), body length (*P* = 0.018; Fig 2C), body barrel (*P* = 0.03; Fig 2D), withers height (*P* = 0.001; Fig 2E), and heart girth (*P* = 0.001; Fig 2F), with higher measures for WM-fed calves than in those fed MR. There were also interactions observed on ADG (*P* = 0.046; Fig 3C) and BW (*P* = 0.023; Fig 3B). The WM-fed calves showed a greater feed efficiency compared to the MR-fed calves (T × P interaction; *P* = 0.001; Fig 3D).

### Sorting activity

**Fig 4:**
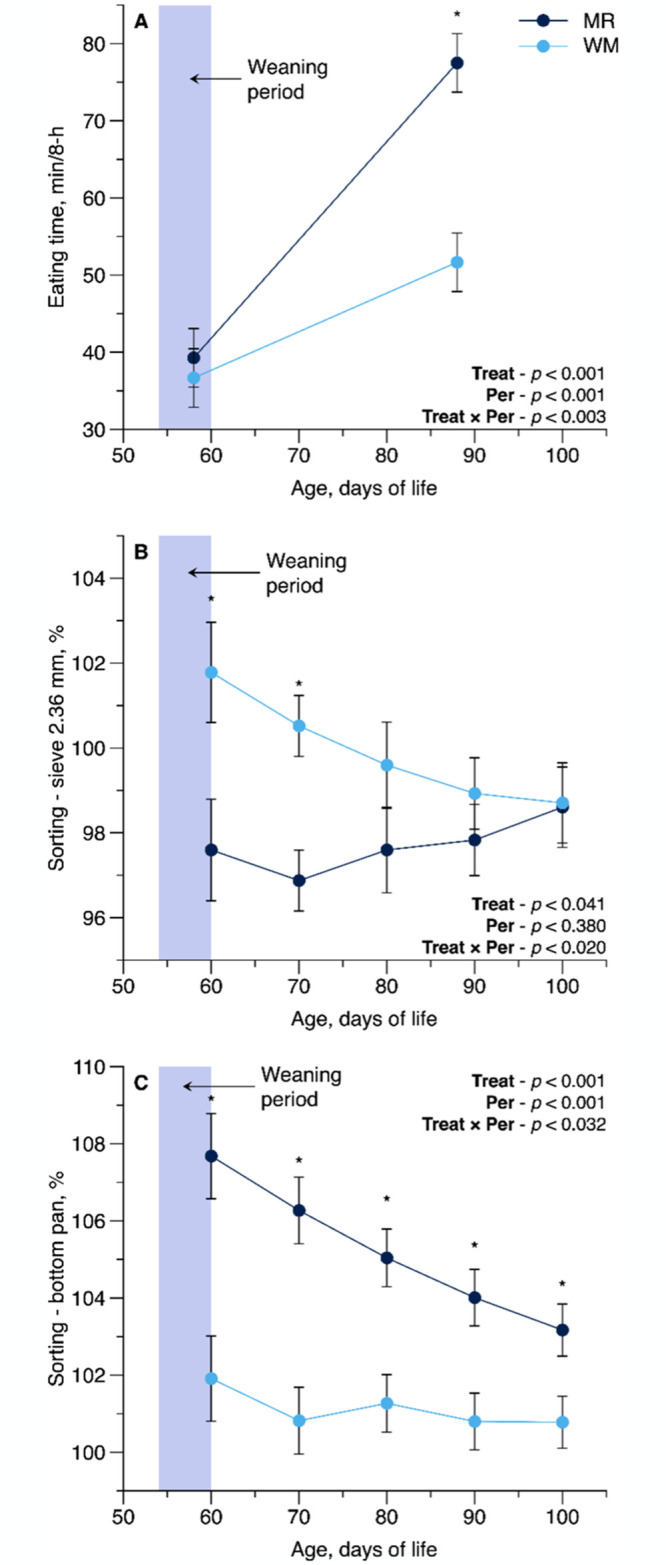
Interaction panel of Treat × Period effect (T × P) for eating time (min/8-h) and sorting index (%) on sieve 2.36 mm and bottom pan as influenced by feeding pasteurized waste milk (WM) or milk replacer (MR) as liquid feeds to newborn Holstein calves (n = 16 per treatment). For each time point, * denotes a significant difference at *P* ≤ 0.05. Error bars represent the standard error of the mean.

The WM-fed calves sorted more against particles retained on the sieve of 0.6 mm (*P* = 0.009) and more for 4.75 mm (*P* = 0.047), and there was an interaction showing less sorting against feed materials retained on the 2.36 mm sieve (*P* = 0.041) and less for the bottom pan (*P* = 0.001) compared with MR-fed calves (Fig 4). Nutrient intake DM from particles retained on the 2.36-mm (*P* = 0.046), and trends on 4.75-mm (*P* = 0.055) and 1.18-mm (*P* = 0.067) sieves were greater in calves fed WM than in those fed MR (Table 4). No effect was observed from particles retained on the 0.6 mm sieve and the bottom pan (Table 4).

**Table 4:** Sorting index^1^ and particle size intake as influenced by feeding pasteurized waste milk or milk replacer to newborn Holstein calves (n = 16 per treatment).

Item	Treatments (T)		SEM	*P*-value		
	MR	WM		T	Period (P)	T × P
Sorting index, %						
4.75 mm	111.73*	113.24*	0.529	0.047	0.001	0.634
2.36 mm	97.70*	99.91	0.726	0.041	0.380	0.020
1.18 mm	95.81*	96.46*	0.490	0.349	0.001	0.480
0.6 mm	94.92*	92.76*	0.547	0.009	0.317	0.325
Pan	105.23*	101.11*	0.655	0.001	0.001	0.032
Starter DM intake, g/d						
4.75 mm	578.13	644.13	23.445	0.055	0.046	0.495
2.36 mm	396.81	445.89	16.745	0.046	0.053	0.625
1.18 mm	900.55	1000.04	37.174	0.067	0.051	0.570
0.6 mm	503.48	544.19	21.080	0.181	0.056	0.516
Pan	267.74	284.59	10.490	0.264	0.048	0.443
NDF intake, g/d						
4.75 mm	111.00	123.67	4.520	0.055	0.046	0.495
2.36 mm	76.19	85.61	3.254	0.046	0.053	0.625
1.18 mm	172.90	192.01	7.089	0.067	0.051	0.570
0.6 mm	96.67	104.48	4.016	0.181	0.056	0.516
Pan	51.41	54.64	1.983	0.264	0.048	0.443

MR = milk replacer; WM = waste milk; DM = dry matter; NDF = neutral detergent fiber; SEM = standard error of the mean.

^1^Sorting % = 100 × (actual particle-size fraction DM intake/predicted particle-size fraction DM intake). Values equal to 100% indicate no sorting, <100% indicate selective refusals (sorting against), and >100% indicate preferential consumption (sorting for). Data are averaged over 10 d for each 10-d period.

*P ≤ 0.05; sorting values differ from 100% using the *t*-test procedure of SAS.

### Diurnal meal pattern and chewing behavior

There was only a trend for T × P interaction on meal frequency regarding eating rate (*P* = 0.095), whereas WM-fed calves had a lower number of eating bouts (*P* = 0.046) but a greater rate of eating of starter feed DM/min (*P* = 0.001) compared with MR fed calves (Table 5). Calves fed WM tended to have longer inter-meal intervals (*P* = 0.097; Table 5) and shorter meal lengths (*P* = 0.027) than calves fed MR. Meal size of starter feed DM/bout (*P* = 0.008) and starter feed DM intake (*P* = 0.007) were also greater in WM *vs*. MR-fed calves. Treatment tended to affect the interval between rumination bouts (*P* = 0.08) with shorter intervals in WM calves compared to MR calves (Table 5).

**Table 5:** Meal and rumination patterns and times devoted to feeding activities as influenced by feeding pasteurized waste milk or milk replacer to newborn Holstein calves (n = 16 per treatment).

Item	Treatments (T)		SEM	*P*-value		
	MR	WM		T	Period (P)	T × P
Meal						
Bouts (frequency)/8 h	5.83	5.08	0.260	0.046	0.011	0.460
Length, min	10.21	8.76	0.444	0.027	0.001	0.455
Interval, min	86.27	101.77	6.303	0.097	0.010	0.247
Eating rate, g of SDMI/min	13.68	42.39	1.841	0.001	0.001	0.095
Meal size, g of SDMI/bout	149.56	207.64	14.652	0.008	0.001	0.154
Rumination						
Bouts (frequency)/8 h	4.02	4.63	0.302	0.168	0.001	0.422
Length, min	18.50	18.32	0.682	0.850	0.001	0.907
Interval, min	123.46	97.43	10.133	0.080	0.001	0.545
Eating time, min	58.39	44.17	2.682	0.001	0.001	0.003
Ruminating time, min	69.58	84.27	4.840	0.036	0.660	0.575
Resting time, min	316.35	309.64	6.055	0.435	0.002	0.226
Drinking time, min	11.09	13.70	1.354	0.184	0.006	0.496
NNOB, min	24.58	28.23	2.433	0.297	0.002	0.457
Standing time, min	169.43	147.03	5.787	0.010	0.215	0.207
Laying time, min	310.57	332.97	5.787	0.010	0.215	0.207

MR = milk replacer; WM = waste milk; SDMI = starter dry matter intake; NNOB = non-nutritive oral behaviors; SEM = standard error of the mean.

There was a T × P interaction effect (*P* = 0.003) on time devoted to eating; WM-fed calves spent less time eating compared with MR-fed calves in the post-weaning (Fig 4A). Time dedicated to rumination was greater in WM-fed calves than in those fed MR (*P* = 0.036). Time dedicated to resting, drinking, and NNOB did not differ between treatment groups. Time dedicated to standing and lying decreased and increased in calves fed WM *vs*. MR, respectively.

### Health

**Fig 5:**
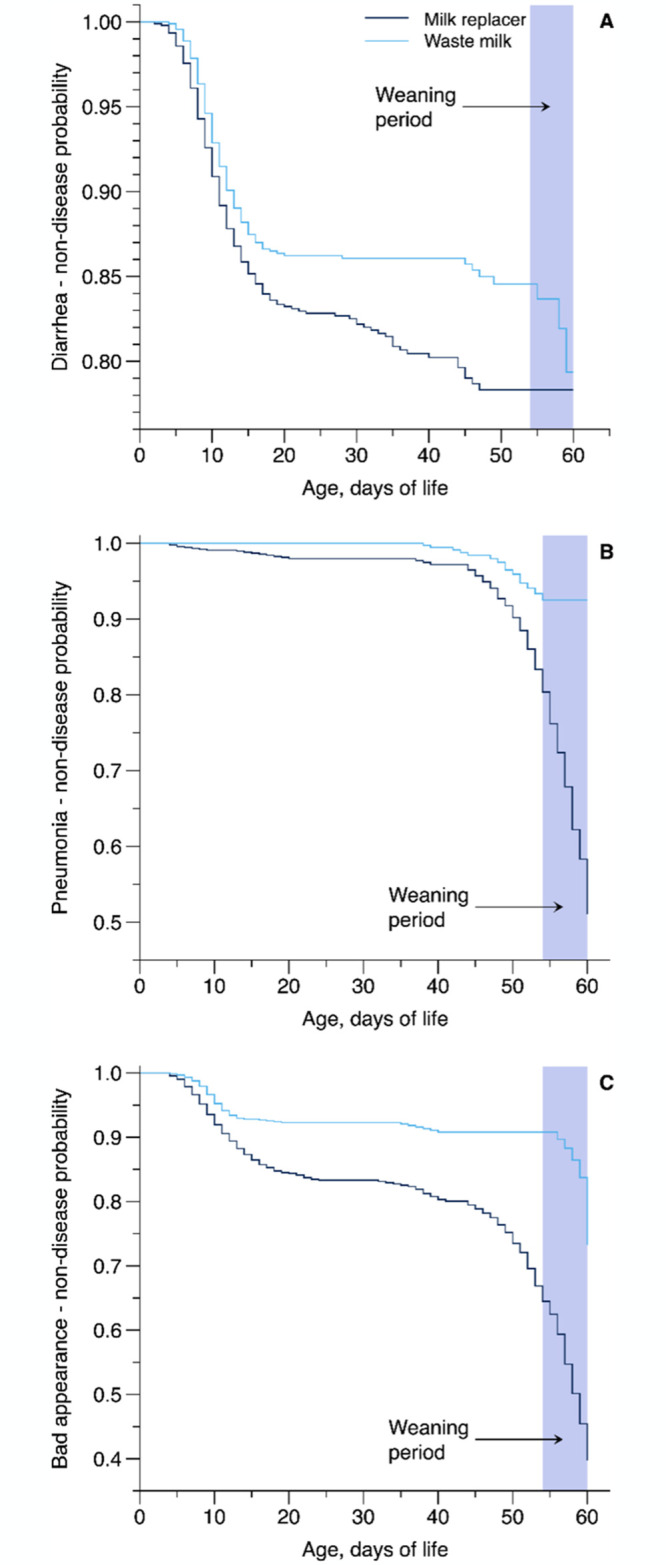
Panel of Kaplan-Meier survival curves showing the non-disease probability of diarrhea, pneumonia, and bad general appearance as influenced by feeding pasteurized waste milk (WM) or milk replacer (MR) as liquid feeds to newborn Holstein calves (n = 16 per treatment).

Table 6 and Fig 5 show the logistic models and non-disease probability of elevated general appearance (≥2), diarrhea (≥3), and pneumonia through the pre-weaning (d 1 to 61) period. Calves fed WM had a lower occurrence of diarrhea (hazard ratio = 1.35; *P* = 0.01; Fig 5A) and pneumonia (hazard ratio = 4.77; *P* = 0.001; Fig 5B) when compared with MR-fed calves. The occurrence of elevated general appearance decreased in WM-fed calves than those fed MR (hazard ratio = 2.79; *P* = 0.001; Fig 5C).

**Table 6:** Logistic model for elevated general appearance (≥2)^1^, diarrhea (≥3)^2^, and pneumonia occurrence before weaning as influenced by feeding pasteurized waste milk or milk replacer to newborn Holstein calves (n = 16 per treatment).

Variable and comparison	Estimate	SEM	*P*-value	Hazard ratio^3^	95% CI	
					Lower	Upper
General appearance (MR *vs*. WM)	1.027	0.133	0.001	2.792	2.152	3.623
Diarrhea occurrence (MR *vs*. WM)	0.300	0.118	0.010	1.350	1.072	1.700
Pneumonia occurrence (MR *vs*. WM)	1.563	0.284	0.001	4.772	2.734	8.329

MR = milk replacer; WM = waste milk; SEM = standard error of the mean; CI = confidence interval.

^1^1 = normal and alert; 2 = ears drooped; 3 = head and ears drooped, dull eyes, slightly lethargic; 4 = head and ears drooped, dull eyes, lethargic; and 5 = severely lethargic [33].

^2^1 = normal; 2 = soft to loosen; 3 = loosen to watery; 4 = watery, mucous, and slightly bloody; 5 = watery, mucous, and bloody [33].

^3^The hazard ratio indicates the probability of having elevated general appearance (≥2), diarrhea (≥3) or pneumonia for the experimental diets (WM *vs*. MR). If the OR is >1, a given liquid feed in the comparison is more likely to have elevated general appearance, diarrhea, or pneumonia than the other liquid feed by a factor of the difference above 1. If the OR is <1, a given liquid feed has a lower probability of occurrence than the other liquid feed.

Table 7 shows the Poisson regression results for days with elevated general appearance (≥2), frequency and duration of diarrhea (≥3), pneumonia, and need for medication. Calves fed WM experienced fewer days (*P* = 0.001) with elevated general appearance compared with calves fed MR. The frequency of diarrhea and pneumonia were not affected by treatment; however, WM-fed calves experienced episodes of shorter duration of diarrhea (*P* = 0.01) and pneumonia (*P* = 0.001) and needed fewer days of veterinary treatment (*P* = 0.001) for pneumonia compared with MR-fed calves.

**Table 7:** Poisson regression for days with elevated general appearance (≥2)^1^, frequency and duration of diarrhea (≥3)^2^ and pneumonia, and medicated days before weaning as influenced by feeding pasteurized waste milk or milk replacer to newborn Holstein calves (n = 16 per treatment).

Item	Treatments (T)		SEM	*P*-value
	MR	WM		T
Days with elevated general appearance (≥ 2)	13.4	4.8	0.091	0.001
Diarrhea				
Number of calves diagnosed at least for once	16/16	16/16	—	—
Frequency, no. of diagnosed times	1.9	1.7	0.186	0.599
Duration, d	10.6	7.9	0.082	0.010
Medication, d	7.0	6.0	0.098	0.267
Pneumonia				
Number of calves diagnosed at least for once	9/16	4/16	—	—
Frequency, no. of diagnosed times	0.8	0.3	0.362	0.169
Duration, d	4.4	0.9	0.188	0.001
Medication, d	4.1	1.6	0.162	0.001

MR = milk replacer; WM = waste milk; SEM = standard error of the mean.

^1^1 = normal and alert; 2 = ears drooped; 3 = head and ears drooped, dull eyes, slightly lethargic; 4 = head and ears drooped, dull eyes, lethargic; and 5 = severely lethargic [33].

^2^1 = normal; 2 = soft to loosen; 3 = loose to watery; 4 = watery, mucous, and slightly bloody; 5 = watery, mucous, and bloody [33].

## Discussion

In the present study, calves fed WM consumed numerically less DM from the liquid feed relative to target levels of 6 kg/d, indicating a marginally greater milk refusal in MR-fed calves (Table 3); however, MR-fed calves consumed greater DM (+28 g/d) and lactose (+63 g/d) but not CP (–7 g/d), ether extract (–41 g/d), and metabolizable energy (–0.12 Mcal/d) from liquid feed diet which partially reflects the higher contents of DM (+0.63%) and lactose (+8.89%) and lower contents of CP (–2.28%), fat (8.06%), and metabolizable energy (–0.43 Mcal/kg of DM) in MR *vs*. WM diet (Table 1), suggesting that MR-fed calves were striving to meet their caloric requirements by increasing the intake of a lesser energy-dense liquid feed, although they failed to achieve it. These results align with recent studies in which the calves that had free-choice access to an MR containing higher fat level consumed less liquid feed than the calves receiving MR containing less fat [11,12]. Additionally, Wilms et al. [36] study showed the number of rewarded visits to the automated milk feeders was 14% greater in calves fed high-lactose-MR than calves fed high-fat-MR, although liquid feeds were provided ad libitum, which resulted in 13% greater milk intake in high-lactose-MR calves. They deduced that the greater number of rewarded visits and the higher MR intake in high-lactose calves could be due to the lower energy density in the high-lactose MR diet compared with high-fat MR.

Feeding WM increased nutrient intake from the starter feed despite consuming less milk. Berends et al. [37] reported no association between energy intake from liquid and energy starter feed. It was reported that feeding liquid feed with similar nutrient composition from non-medicated all-milk protein MR (26.0% CP and 31.0% fat, on a DM basis; diluted to 12.5% DM) or pasteurized WM containing traces of anti-microbial (28.4% CP and 30.1% fat, on a DM basis) increased the starter feed intake in WM-fed calves [38]. Infer of any study comparing the starter feed intake between dairy calves feeding on WM and MR containing different energy sources (fat *vs*. lactose) but similar in CP:ME ratio. Feeding WM [15] or MR [17] containing antibiotic residues did not affect starter feed intake in milk-fed Holstein dairy calves. According to Hill et al. [39], pre-weaning (28-d-period) starter feed intake responded quadratically to the level of fat, being lowest at 14 and 23% fat in MR; however, lard, a poorly digested fat, was the predominant fat source in the MR. Yohe et al. [40] studied four different rates of fat inclusion at the expense of lactose in MR and reported no difference in starter feed intake during pre- and post-weaning periods. Amado et al. [9], partially exchanging lactose for fat in MR, reported no changes in starter feed intake during the entire 70-d-period of the experiment; however, an increase in starter feed intake was observed in calves fed low-fat high-lactose MR during the weaning period (wk. 7 to 9), opposing the finding in the present study. Berends et al. [11], exchanging lactose for fat in MR, reported increased starter feed intake during wk. 2 to 7 of the study in calves fed ad libitum, a high-fat, low-lactose MR. The source and the macronutrient composition of the liquid feed (WM *vs*. MR) may also affect blood concentrations of growth factors [41], which play an important role in the development of the small intestine and rumen [35], thereby encouraging starter feed intake and digestion [35,42].

The higher starter feed nutrient intake in calves fed WM than MR was due to higher nutrient intake from particles retained on the sieves of 4.75-, 2.36-, and 1.18-mm (Table 4). It has been reported that dairy calves can sort feed particles at an early life, and sorting behavior in calves may depend on previous experience and dietary requirements [3,28,29,32]; however, higher sorting for particles retained on the 4.75 and feed materials on the pan in MR-fed calves was counteracted by sorting against particles retained on the 2.36-mm and 0.6-mm sieves and therefore, those calves did not have comparable starter feed intake to WM-fed calves, suggesting a long-lasting effect of the chemical composition of the liquid feed on sorting behavior in dairy calves. Thus, the mechanism(s) through which the energy source (fat *vs*. lactose) in liquid feed (WM *vs*. MR) affects the sorting behavior in dairy calves needs to be further clarified.

Despite higher meal length and shorter (a tendency) inter-meal interval and thereby higher meal frequency and time dedicated to eating in MR-fed calves, WM-fed calves, due to higher eating rate, had a higher meal size and therefore an increased starter feed intake, suggesting that the strive for increasing starter feed intake was failed in MR-fed calves (Table 5 and Fig 4A). Rumination activity is mainly affected by NDF intake (Table 3) [29]; therefore, the higher NDF intake from particles retained on the 4.75-, 2.65-, and 1.18-mm sieves and a tendency for shorter rumination interval in WM-fed calves could correspond to an increase in time devoted to rumination in WM *vs*. MR-fed calves. The reason(s) for longer standing time in MR-fed calves is (are) unclear; however, this phenomenon might be related to more time devoted to eating during the post-weaning period (74.1 *vs*. 51.7 min/8 h), which is a sign of hunger [43].

Glucose utilization and FA oxidation are important metabolic feedback signals regulating feed intake [22] and feeding behavior [9,12,21]. Despite the higher palatability of high-fat diets, the difference between post-prandial fat and carbohydrate metabolism may contribute to the higher feed intake in high-fat *vs*. high-lactose diets. The extent to which the feed intake is reduced after a meal is usually corresponding to about 40–70% of the consumed energy and dependent on the source of energy (fat or carbohydrate), with fats being generally less effective than carbohydrates [22], suggesting that the short-term satiating effect of consumed fats, calorie for calorie, is lower than that of carbohydrates. This may be attributed to the incomplete oxidation of FA from consumed fat as long as other fuels are available [22]. Energetically, less energy is required to deposit consumed fat as body fat than other nutrients in the adipose tissue, and the glycogen deposits are much smaller than the fat deposits. Therefore, most of the fat consumed seems to be deposited, whereas carbohydrates are predominantly and rapidly oxidized. Accordingly, the respiratory quotient increases even in response to high-fat diets [44], increasing feed intake [45]. Starter feed intake was higher during the weaning period (wk. 7 to 9) for high-lactose *vs*. high-fat MR-fed calves, but it was equal to the whole experiment [9]. During the weaning period (wk. 5 to 8), high-lactose MR-fed calves had approximately 4% and 41% more rewarded and unrewarded visits, respectively, to the automatic feeder with no changes in starter feed intake [12], suggesting that calves fed high-lactose MR were unable to compensate for the reduced allowance of liquid feed and energy, and showed more hunger-related behaviors [43]. Interestingly, despite potentially experiencing greater hunger, MR-fed calves exhibited lower eating rates and meal sizes compared to the WM-fed group. This apparent contradiction highlights the complexity of calf feeding behavior during the weaning period and the need for more detailed behavioral analysis to reveal the main mechanisms involved.

As for growth performance (Table 3), some researchers observed that calves fed with WM had higher weight gain than those fed with MR [7,8,38], whereas others did not observe any differences in weight gain when fat replaced lactose as an energy source in MR-fed calves [9,11,12]. The growth-promoting effect of WM might be attributed to the higher starter feed intake [38], higher nutrient density [7,8], or antibiotic residues in WM. The growth-promoting effect of antibiotics is attributed to altering intestinal flora for promoting a healthier intestinal environment [46] or preventing immune functions to save energy for growth purposes [47]. However, when liquid feed containing subtherapeutic antibiotics was fed to dairy calves, neither the starter feed intake nor growth performance was affected [15–17], suggesting that antibiotic residues in WM are not a factor impairing the growth of calves. The improved feed efficiency in WM-fed calves is in line with the findings of Maynou et al. [38] and might be attributed to the greater bioavailability of nutrients in WM compared with MR [2] or the relative decrease in Bacteroidetes phylum and increase in Firmicutes in the gastrointestinal tract [38]. There was a positive relation between the increase in the intestinal Firmicutes and the capacity to harvest energy from the diet, thereby improving feed efficiency in pigs [48] and steers [49]. In the present study, the potential reasons for higher growth rate and skeletal and BW gains in calves fed WM *vs*. MR were due to the higher total nutrient intake and feed efficiency.

As observed in the non-disease probability analysis, the MR-fed calves had a higher probability of having elevated general appearance, diarrhea, and pneumonia (Table 6 and Fig 5). No difference was observed between the treatment groups for frequency and medication days of diarrhea; however, WM-fed calves had fewer days (2.7 d) with diarrhea. The number of days with pneumonia (3.5 d, but not frequency) and administration of medication (2.5 d) were also greater for calves fed MR compared with calves fed WM (Table 7). In the present study, day-to-day variation in WM nutrient composition did not adversely affect the susceptibility, duration, and medication administration for diarrhea and pneumonia relative to MR with a more consistent nutrient composition, which aligns with the findings of Godden et al. [8]. Several possible explanations exist for shorter days with diarrhea and pneumonia or shorter medicated days for pneumonia in WM-fed calves. First, the higher nutrient intake in WM-fed could improve the immune function; anabolic hormones, provided directly by WM or elevated in blood indirectly by higher nutrient intake [40], integrate the immune system’s growth, maintenance, repair, and function [8]. Second, the WM primarily comprises colostrum plus mastitis milk; thus, immunoglobulins and many bioactive molecules in WM may decrease the calf’s neonatal susceptibility to diarrhea and pneumonia [1–3]. Furthermore, because milk fat is caught in the abomasal curd, a continuous release of these anti-microbial FA could occur throughout the day [8]. On the other hand, the delayed development of the small intestine in newborn calves fed an MR diet is well documented [35,41,50,51]. Since liquid feed is the sole nutrient source until calves begin consuming solid feed, and the small intestine is the primary site for liquid feed digestion, this delay can impair nutrient absorption and digestion. As a result, it may negatively impact calf growth and health during the early weeks of life [35]. Moreover, the effect of higher fat content inclusion in MR (≥23%) on fecal consistency improvement [9] and reduction of therapeutic interventions [11] or mortality [5] in newborn calves are proofs of the substantial role of dietary fat from liquid feed on young calves’ health [52]. Therefore, higher fat density in WM (>24%) versus lower density in MR treatment (<17%; Table 1) in the present study could explain the association between lower events of diarrhea and pneumonia and greater fat intake (>40%; Fig 1) and body development in WM-fed calves compared with MR-fed calves during the preweaning period.

The effect of antibiotic residues in WM or MR on health events in dairy calves is controversial. For example, Langford et al. [15] reported that the penicillin content of WM did not affect the incidence of diarrhea in calves fed ad libitum; they associated the lack of effect of antibiotics to the high plane of nutrition through supplying unlimited liquid feed. Thames et al. [16] observed no effect on health events when calves received MR that contained therapeutic or sub-therapeutic dosages of tetracycline and neomycin and attributed this effect to good sanitation and management practices as well as feeding a more nutrient-dense diet with higher intake relative to most previous studies. In contrast, Berge et al. [53] reported that antibiotic addition to WM was associated with a higher percentage of days with diarrhea in calves compared with no antibiotic addition, while respiratory scores were similar between the two treatments, highlighting the potential negative effects of antibiotics on the gastrointestinal microbiome. While the nutritional status and overall health of calves may outweigh the benefits of subtherapeutic antibiotic treatment [16,17], the potential advantages of antibiotic residues in WM fed to calves should not be overlooked, especially given that the MR used in the current study was antibiotic-free.

The anti-microbial levels, types, and duration in WM can vary significantly depending on the treatments used for cows on a given farm. However, the findings across various studies remain inconsistent. For instance, Wray et al. [54] observed no significant difference in the anti-microbial resistance of fecal *Escherichia coli* between calves fed WM and those fed MR. In contrast, Maynou et al. [55] demonstrated that feeding WM increased the prevalence of resistant bacteria in both the gut and nasal microbiota of calves, as seen with *E*. *coli* and *Pasteurella multocida* isolates from fecal and nasal swabs, respectively. However, this study also found high levels of multidrug resistance in both WM- and MR-fed calves. Li et al. [17] further explored the effects of specific antibiotics in MR, reporting that the frequency of diarrhea in calves fed MR containing penicillin, streptomycin, tetracycline, and ceftiofur was comparable to those fed antibiotic-free MR, except for a higher incidence in the antibiotic-free group during the fourth week of life. Lastly, it has also been reported that feeding liquid feed with a lower osmotic value, caused by fat replacing lactose as the energy source in the MR, may positively affect the calf’s health by diminishing the occurrence of events related to the respiratory system [11] by decreasing the respiratory quotient and therefore, a relative decrease in the amount of carbon dioxide (CO_2)_ produced which reduces the respiratory burden to eliminate CO_2_ and the amount of energy spent on respirations [56] or by increasing fecal consistency before weaning [9] through reducing gut permeability [10].

## Conclusion

At constant CP:ME ratio but from different sources (fat *vs*. lactose), feeding WM in contrast to MR increased the starter feed intake by an increase in eating rate and meal size as well as increased selective consumption of the particles that retained on sieves of 4.75-, 2.36-, 1.18-mm and bottom pan. The present results showed that feeding WM increased the growth rate, final BW, and body frame gain of the calves; this was probably because of increased total nutrient intake, decreased susceptibility to diarrhea and pneumonia, and fewer days with elevated general appearance, diarrhea, or pneumonia incidence. Overall, feeding WM may be recommended over MR due to its positive effects on growth and health, as well as reducing the hearing costs of dairy calves.

## Supporting information

S1 Data (XLSX)
